# Minimum variance rooting of phylogenetic trees and implications for species tree reconstruction

**DOI:** 10.1371/journal.pone.0182238

**Published:** 2017-08-11

**Authors:** Uyen Mai, Erfan Sayyari, Siavash Mirarab

**Affiliations:** 1 Dept of Computer Science and Engineering, University of California at San Diego, San Diego, CA, United States of America; 2 Dept of Electrical and Computer Engineering, University of California at San Diego, San Diego, CA, United States of America; Wilfrid Laurier University, CANADA

## Abstract

Phylogenetic trees inferred using commonly-used models of sequence evolution are unrooted, but the root position matters both for interpretation and downstream applications. This issue has been long recognized; however, whether the potential for discordance between the species tree and gene trees impacts methods of rooting a phylogenetic tree has not been extensively studied. In this paper, we introduce a new method of rooting a tree based on its branch length distribution; our method, which minimizes the variance of root to tip distances, is inspired by the traditional midpoint rerooting and is justified when deviations from the strict molecular clock are random. Like midpoint rerooting, the method can be implemented in a linear time algorithm. In extensive simulations that consider discordance between gene trees and the species tree, we show that the new method is more accurate than midpoint rerooting, but its relative accuracy compared to using outgroups to root gene trees depends on the size of the dataset and levels of deviations from the strict clock. We show high levels of error for all methods of rooting estimated gene trees due to factors that include effects of gene tree discordance, deviations from the clock, and gene tree estimation error. Our simulations, however, did not reveal significant differences between two equivalent methods for species tree estimation that use rooted and unrooted input, namely, STAR and NJst. Nevertheless, our results point to limitations of existing scalable rooting methods.

## Introduction

Commonly-used models of sequence evolution, such as GTR [1], are time reversible and can therefore be used to reconstruct *unrooted* phylogenetic trees. The correct placement of the root is often of intrinsic interest as evident by long debates on the correct rooting of the universal tree-of-life [2–7], and other major groups (e.g., [5, 8, 9]). Moreover, the knowledge of the root is often needed for downstream applications of phylogenetic trees, such as ancestral state reconstruction [10], comparative genomics [11], taxonomic profiling of metagenomic samples [12, 13], and dating.

Several approaches have been proposed for this long recognized issue [14]. The current prevailing practice is to simply use outgroups [10]. An outgroup is a species known *apriori* to be outside the group of interest (referred to as the ingroup). Outgroup selection is an art that requires balancing two opposite goals; the outgroup needs to be divergent enough from the ingroup to make its outgroup status unambiguous, but at the same time not so distant that strong long branch attraction [15–17] negatively impacts the resolution of the ingroup, the placement of the outgroup, or both [18–22]. Nevertheless, several studies have found outgroups to be competitive with more complex methods [23, 24] that use evidence from molecular data for rooting.

At one end of the spectrum, rooting an unrooted tree is trivial when the rooted tree is ultrametric (i.e., all leaves are equidistant to the root). Only one rooting of an unrooted tree can create an ultrametric tree, and that rooting can be obtained by midpoint (MP) rooting; i.e., root the tree at the middle point of the longest path between any two leaves of the tree. A phylogenetic tree with branch lengths measured in the expected number of mutations will be expected to be close to ultrametric if mutations follow a strict molecular clock (i.e., rates of mutation are constant). When a strict molecular clock is not followed in the data, one can still use the midpoint rooting, hoping that divergences from a strict clock are small and that midpoint rooting can still be a good proxy for the correct root [25]. At the other end of the spectrum, non-reversible models of sequence evolution, such as the General Markov Model [26–28], or those that incorporate nonstationarity [29, 30], can be used to infer a rooted tree from the data; however, these methods have not yet enjoyed broad application because of statistical issues related to model complexity and lack of scalability to large datasets (but see [31] for recent advances).

Despite the long history of thinking about tree rooting, we believe the question should be revisited in the phylogenomic era. The potential for discordance among gene trees and incongruence with the species tree due to factors such as incomplete lineage sorting (ILS) is now well-understood [32–34] and many empirical analyses strive to account for it [35–39] (but see [40–43] for the ongoing debate on this issue).

Rooting phylogenies needs fresh thinking in the phylogenomic area for several reasons. Firstly, an outgroup is a species believed to be outside the ingroups in the true species tree; however, depending on how the outgroup is chosen, its true position may or may not be outside the ingroups in every single gene tree. As an example, according to the multi-species coalescent model [44], an outgroup separated from the ingroups by a branch of length 2 in coalescent units [33] (corresponding to 8 millions years assuming a diploid effective population size of 200,000 and a generation time of 10 years) is expected to be mixed with the ingroups in 9% of genes only because of ILS effects and optimistically assuming that all basal branches of the ingroups are so long that only two lineages have to coalesce in the branch below the root. Thus, even if outgroups are reliable methods of rooting a species tree, they may fail to root every gene tree accurately. A second reason to revisit rooting is related to the practice of species tree estimation. The most scalable pipeline for estimating a species tree first estimates a set of gene trees and then uses a “summary method” to combine the estimated gene trees to reconstruct a species tree. Some summary methods (e.g., MP-EST [45], STAR/STEAC [46], and GLASS [47]) rely on rooted input gene trees, while more recent methods (e.g, ASTRAL [48, 49] and NJst [50, 51]—also known as USTAR/NJ [52]) can combine unrooted gene trees. Even though the question has never been directly addressed before, the accuracy of methods based on unrooted trees tends to be superior to rooted trees on simulated and empirical data [41, 48–50, 53]. It remains to be tested if these trends relate to incorrect rooting of gene trees, as suggested by some studies [41]. Finally, reconciliation between gene trees and a species tree may provide a way to root them. Gene duplication history, the number of deep coalescences, and distributions of unrooted gene trees have all been used to root gene trees, species trees, or both [54–58]. However, in this manuscript, we will focus on rooting gene trees individually and not collectively or with reference to a known species tree.

Beyond phylogenomics, the ever-expanding size of phylogenetic trees is another factor that should be considered in discussions of rooting. Trees with thousands of leaves are routinely inferred and used currently, and trees with many hundreds of thousands of leaves are also in use [5, 59–61]. We should ask whether rooting such large trees with existing methods is computationally feasible, and if so, whether they are accurate.

In this paper, we address the problem of rooting large phylogenomic datasets. We introduce a new rooting method that minimizes the variance of the root to tip distances. We implement our new method, called min-var (MV) rooting, in an algorithm that scales linearly with the tree size, just like the MP rooting (note that the term minimum variance used here does not relate to statistical minimum variance estimators). We compare MV and MP with outgroup (OG) rooting under a wide range of conditions where gene trees and the species tree can be discordant, with a range of dataset sizes, with several ways of choosing an outgroup, and with various levels of divergence from a strict clock. We then go on to compare several species tree reconstruction methods, including those that use inferred unrooted trees, or trees rooted using the three rooting approaches. Our rooting tool is publicly available at https://uym2.github.io/MinVar-Rooting/.

## Materials and methods

### Min-var (MV) rooting

#### Notations and definitions

Let an unrooted tree be represented as a connected acyclic undirected graph *G* = (*V*, *E*), and let each edge *e* = (*u*, *v*) ∈ *E* be weighted by a length *w*_*e*_. To root *G* at an edge *e* = (*u*, *v*) ∈ *E* and a position *x* ≤ *w*_*e*_ from *u*, we first divide *e* to two edges by a vertex *p* and replace *e* with edges (*p*, *u*) and (*p*, *v*) with lengths *x* and *w*_*e*_ − *x*, respectively. Then, we convert *G* to a directed graph by pointing all its edges away from *p*. The resulting graph is a rooted tree, *T*, and is a *rooting* of *G*.

We use the following notations for a rooted tree *T*. Each node *u* in *T*, except the root *r*_*T*_, has a parent, *p*(*u*), and the child set of a node *u* is denoted by *c*(*u*). A node *u* is either *internal* and has two or more children or is a *leaf* and has no children. The set of leaves is denoted by *L* = {1…*n*}. For any node *u*, we denote the length of the edge (*p*(*u*), *u*) by *e*_*u*_. For each point *p* on this edge (including *u*), we let *Cld*(*p*) denote the set of leaves descending from node *u* and |*p*| is used for the size of *Cld*(*p*). For two points *p* and *p*′, potentially on different edges, we let *d*(*p*, *p*′) denote the total length of the *undirected* path from *p* to *p*′, and use *d*_*i*_(*p*) = *d*(*i*, *p*) as a shorthand for *i* ∈ *L*. We set *mean*(*p*) = 1/*n*∑_*i*∈*L*_ *d*_*i*_(*p*), *var*(*p*) = 1/*n*∑_*i*∈*L*_(*d*_*i*_(*p*) − *mean*(*p*))^2^, *SI*(*p*) = ∑_*i*∈*Cld*(*p*)_ *d*_*i*_(*p*), and *ST*(*p*) = ∑_*i*∈*L*_ *d*_*i*_(*p*).

We call a *p*_0_ a *local MV* of tree *T* if and only if for any point *p* and *x* = *d*(*p*_0_, *p*),

$$\begin{array}{c} \lim_{x\to 0}\frac{var\left(p\right)-var\left(p_{0}\right)}{x}=0 \end{array}$$ (1)

and the second derivative of *var*(*p*_0_) is non-negative (i.e., *var*(*p*) > *var*(*p*_0_)).

The *global MV* of a tree is a point *p*_0_ that has the minimum *var*(*p*_0_) among all positions on all branches of the tree. Unless otherwise specified, we use the terminology *MV* to refer to the *global MV*.

A point *p* is said to be a *balance point* of *T* if the average of tip distances to *p* are equal on any two sides of *p* in the unrooted version of *T*; that is, *p* is a balance point if

$$\begin{array}{c} \frac{1}{\left|u\right|}\sum_{i\in Cld(u)}d_{i}\left(p\right)=\frac{1}{n-|u|}\sum_{i\notin Cld(u)}d_{i}\left(p\right) \end{array}$$ (2)

for all ways of choosing *u* such that *p* is on the edge (*p*(*u*), *u*) (including both ends).

#### Problem statement

MP rooting can be framed as an optimization problem that seeks the rooting position that minimizes the maximum distance from any leaf to the root. Our proposed approach, MV rooting, is based on a similar idea, but minimizes the variance instead of the maximum.

The MV problem is: *Given* an unrooted tree *G*, *find* a rooting T* of *G* such that

$$\begin{array}{c} T^{*}=\underset{T}{\mathrm{argmin}}var\left(r_{T}\right)\;\;. \end{array}$$ (3)

Thus, we seek the root that minimizes the variance of root to tip distances.

#### Motivation for MV rooting

We start with the following propositions (proofs are shown in S1 Appendix)

Proposition 1. *A point p on tree T is a local MV if and only if it is a balance point*.

Based on Proposition 1, we refer to local MV and balance point interchangeably.

Proposition 2. *Any tree has at least one local MV*.

Proposition 3. *The global MV of any tree is one of its local MVs*.

When the strict molecular clock is followed, the true rooted phylogenetic tree is ultrametric with zero root-to-tip distance variance. For ultrametric trees, only the true rooting position is a balance point, and therefore, the tree has a unique local MV at the correct root, which is also its global MV (Proposition 3). Since local MVs are also balance points, they provide a natural choice for rooting when there are randomly distributed deviations from the molecular clock. Among several local MVs, the global MV also minimizes the total variance, and arguably is the best choice. We now describe a simplified model under which we can prove that MV is in expectation the correct root.

*Random deviations model*: Consider a model where a rooted tree *T* is generated from an ultrametric tree *T*_0_ by multiplying the length of each edge (*u*, *v*) by a random variable *α*_*v*_ drawn from any distribution with support [1 − *ϵ*, 1 + *ϵ*] and expected value 1. Let *h* be the height of *T*_0_ and *r* be the position of the true root on *T*, which it inherited from *T*_0_. We have the following two propositions (proofs are shown in S1 Appendix).

Proposition 4. *Let p denote the global MV of T*. *If*

$$\begin{array}{c} \epsilon \leq \min_{w\in c(r)}(\frac{e_{w}}{\frac{n}{n-|w|}h+e_{w}}) \end{array}$$

*then there exists a child w of r such that p* ∈ *e* = (*r*, *w*)

Following Proposition 4, the global MV is guaranteed to be on one of the adjacent edges of *r* if *ϵ* is sufficiently small. Note that the restriction on *ϵ* is a sufficient but not a necessary condition. Regardless of the value of *ϵ*, we can also show the following.

Proposition 5. *When the global MV is on one of the adjacent edges of r*, *let a random variable X indicate the distance of the global MV to the root*; *then*, *E*(*X*) = 0.

Corollary 1. *Under our random deviations model where deviations from the strict molecular clock are independent and bounded*, *the MV rooting will find the correct branch*, *and in expectation*, *will also have zero distance on that branch to the correct rooting position*.

Although the random deviations model considerably simplifies real biological processes, it is useful in motivating the MV rooting approach in general.

**Algorithm 1** Linear time MinVar rooting algorithm

Function MinVarRoot(*T*)

 For node *u* in pre-order(*T*)      # Top-down traversal

  Compute *d*(*u*, *r*_*T*_) = *d*(*p*(*u*), *r*_*T*_) + *e*_*u*_ for *i* ∈ *L*∖{*r*_*T*_}

 *minvar* ← *σ*^2^({*d*_*i*_(*r*_*T*_)|*i* ∈ *L*})      # *σ*^2^ is variance

 For node *u* in post-order(*T*)      # Bottom-up traversal

  Store |*u*| = 1 if *u* ∈ *L*, else |*u*| = ∑_*v*∈*c*(*u*)_|*v*|

  Store *SI*(*u*) = 0 if *u* ∈ *L*, else *SI*(*u*) = ∑_*v*∈*c*(*u*)_(*SI*(*v*) + *e*_*v*_|*v*|)

 *globalMV* ← *r*_*T*_

 For node *v* and *u* = *p*(*v*) in pre-order(*T*)      # Top-down traversal

  Compute and store *ST*(*v*) using Eq 6

  Compute and store *var*(*v*) using Eq 4

  Compute x* using Eq 7 and call the corresponding point p*

  Compute *var*(p*) using Eq 4

  if *minvar* > *var*(p*)

   *minvar* ← *var*(p*)

   *globalMV* ← p*

 reroot *T* at p*

#### The MV rooting algorithm

The algorithm is based on the following proposition (proof is shown in S1 Appendix)

Proposition 6. *Let p be a point on an edge* (*u*, *v*) *of tree T with distance d*(*p*, *u*) = *x*. *If we let p vary along edge* (*u*, *v*) *and consider var*(*p*) *as a function of variable x with parameters u and v*, *then*:

$$\begin{array}{c} var\left(p\right)=var\left(x;u,v\right)=\left(1-\beta^{2}\right)x^{2}+(\alpha -\frac{2ST(u)\beta }{n})x+var\left(u\right) \end{array}$$ (4)

*in which*

$$\begin{array}{c} \alpha =\frac{2ST\left(u\right)-4(SI\left(v\right)+|v|e_{v})}{n}\;\;\;\;and\;\;\;\;\beta =1-\frac{2|v|}{n} \end{array}$$ (5)

To find the MV root, we first arbitrarily root the unrooted tree at *r*_*T*_ to get a rooted tree *T*. We then use Algorithm 1 to traverse *T* three times to search for local MVs. At the end, we select the local minimum with the lowest variance value as the global MV.

*Traversal 1 and 2 (Preprocessing)*: In the first top down traversal, we trivially compute the distance to root (i.e., *d*(*u*, *r*_*T*_)) for all nodes of the tree, and then simply compute the variance of root-to-tip distances. Next, in a post-order traversal, for each node *u*, we compute the size of its clade (i.e., |*u*|) and the sum of distances to the tips in its clade (i.e., *SI*(*u*)), both of which are simple to compute.

*Traversal 3*: The final top-down traversal finds the local MV along each edge (*u*, *v*) if it exists, and records the local MV with the minimum root-to-tip variance as the global MV. We set *ST*(*r*_*T*_) = *SI*(*r*_*T*_) and for other nodes we compute and store:

$$\begin{array}{c} ST\left(v\right)=ST\left(p\left(v\right)\right)+\left(n-2|v|\right)e_{v}. \end{array}$$ (6)

According to Proposition 6, for any point *p* along the edge (*u*, *v*) with *x* = *d*(*u*, *p*), we can compute *var*(*p*) (the variance of root-to-tip distance if we root at *p*) using Eq 4. Let *a* = (1 − *β*^2^), $b=(\alpha -\frac{2ST(u)\beta }{n})$, and c = *var*(*u*); Eq 4 is a standard quadratic function *ax*^2^ + *bx* + *c* with *a* > 0 (because |*β*| < 1) and with the restriction *x* ∈ [0, *e*_*v*_]. Thus, *var*(*p*) is minimized on a point p* with distance x* from *u* where:

$$\begin{array}{c} x^{*}=\{\begin{array}{cc} \frac{-b}{2a}, & \text{if}\;\frac{-b}{2a}\in \left[0,e_{v}\right]\\ {\mathrm{argmin}}_{x\in \{0,e_{v}\}}\left(var\left(x;u,v\right)\right), & \text{otherwise} \end{array} \end{array}$$ (7)

and p* is a local MV of *T* only if $x^{*}=\frac{-b}{2a}$. Since we compute this for all edges, at the end, we have all local MVs and their corresponding root-to-tip variance; we simply select the local MV point that has the lowest variance and reroot *T* on that point. Derivation of Eq 6 and the proof for Proposition 6 are shown in S1 Appendix.

Theorem 1. *Algorithm 1 is guaranteed to find the global MV*.

*Proof*. It is clear that Eq 7 minimizes Eq 4 given the constraint *x* ∈ [0, *e*_*v*_] (recall that the second derivative *a* > 0) and thus finds local MV points. According to Proposition 6, Eq 4 gives the correct variance of root-to-tip distances for any point on the tree. By the definition of global MV and Propositions 2 and 3, the global MV *p* is always the local MV with the minimum *var*(*p*). Because Algorithm 1 checks all edges for all local MVs and compute root-to-tip variance at all of those points, it guarantees to find the correct global MV.

Proposition 7. *The running time of Algorithm 1 scales linearly with the number of leaves in the tree*.

*Proof*. Algorithm 1 visits each edge in *T* exactly three times, each of which involves only constant time operations. After the rooting position is found, rerooting the tree also takes no more than linear time assuming that the tree is represented with the usual pointer structure. Thus, the overall time complexity of Algorithm 1 is O(n).

Similar to MV, MP rooting can be done in linear time using two tree traversals (Algorithm 2). Interestingly, at least one phylogenetic package in common use, Dendropy, seems to have opted not to implement this simple algorithm, and instead uses an approach that scales quadratically with *n* (our attempt to use ape [62] failed). We re-implemented MP using the Dendropy package to solve this shortcoming.

### Experimental design

#### Simulated datasets

We study four simulated datasets, including two that were previously published. One of the published datasets, RNASim [63], includes only one gene tree and is used here only to evaluate the scalability of rooting methods. The other datasets all use SimPhy [64] to generate gene and species trees under the multi-species coalescent (MSC) model [44] and heterogeneous parameters. We then used Indelible [65] to simulate nucleotide sequence evolution on gene trees according to the GTR+Γ model with varying sequence length and different sequence evolution parameters (Supplementary methods in S1 Appendix). Then, FastTree2 [66] was used to estimate gene trees based on the GTR+Γ model.

**Algorithm 2** Linear time midpoint rooting algorithm.

Function MidpointRoot(*T*)

 For node *u* in post-order(*T*)      # Bottom-up traversal

  *MI*(*u*) ← max({*MI*(*v*) + *e*_*v*_|*v* ∈ *c*(*u*)})

 *MO*(*r*_*T*_) ← 0

 For node *v* in pre-order(*T*)      # Top-down traversal

  *MO* ← max({*MO*(*p*(*v*))}∪{*MI*(*s*) + *e*_*s*_|*s* ∈ *c*(*p*(*v*)) − {*v*}})

  x* ← (*MI*(*v*) − *MO* + *e*_*v*_)/2

  if x* ≥ 0 and x* ≤ *e*_*v*_

   reroot *T* at (*u*, *v*) with distance x* from *u* and return

  *MO*(*v*) ← *MO* + *e*_*v*_

The three main datasets with species trees and gene trees are:

- D1—30-taxon heterogeneous dataset: Here, the number of ingroup species was fixed to 30. We simulated 100 replicates, each with a different species tree and 500 gene trees. This dataset is used for extensive analysis of all methods.
- D2—Large heterogeneous dataset: This dataset includes two subsets, one with 2000 and another with 5000 taxa, and is used for testing performance on large datasets. For both datasets we created 20 replicates with different species trees and 50 gene trees.
- D3—ASTRAL-II dataset: We reused a previously published dataset [49] to investigate performance for intermediate number of species (i.e, 10, 50, 100, 200, 500, and 1000).

**Fig 1:**
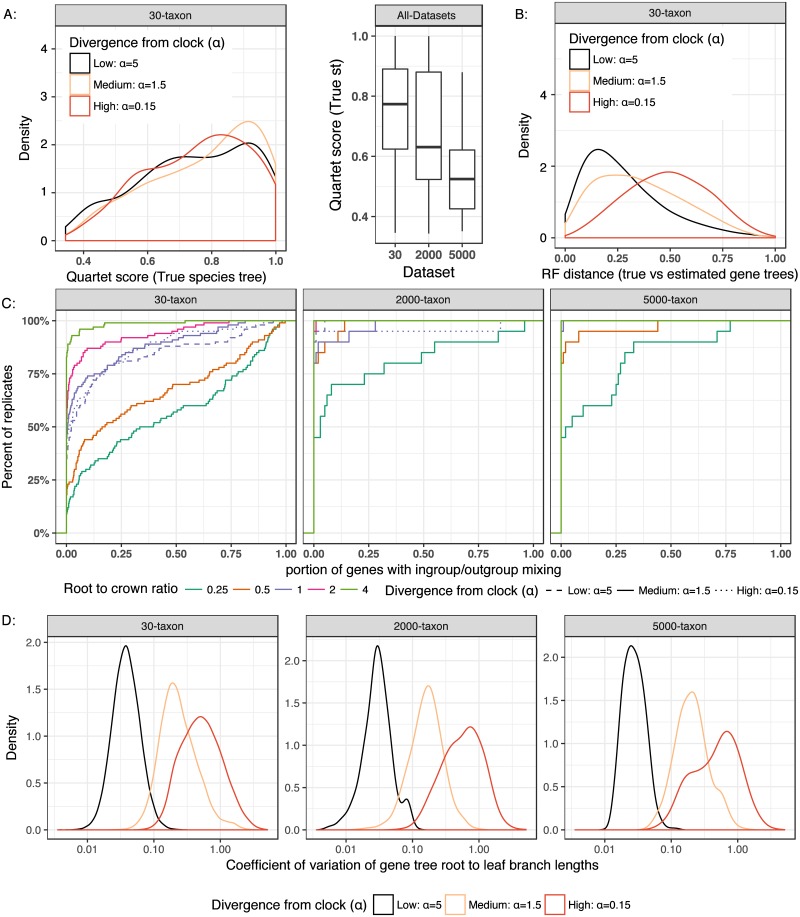
Properties of simulated datasets D1 and D2. A: The level of ILS, measured by the quartet score of true species tree with respect to true gene trees with R/C = 1 for (left) the D1 dataset, broken down by the clock divergence parameter and (right) both D1 and D2 datasets. B: gene tree estimation error, measured as the normalized Robinson-Foulds (RF) distance [67] between true and estimated gene trees for the D1 dataset with R/C = 1 and varying clock divergence parameters. C: The empirical cumulative distribution for the proportion of true gene trees where the outgroup species is not an outgroup; thus, each point (*x*, *y*) on a line indicates that *y* out of 100 replicates had *at most* *x* × 500 true gene trees where the species tree outgroup was not the gene tree outgroup. Boxes correspond to the three datasets with different sizes. D: The ratio between standard deviation to mean (i.e., coefficient of variation) of root to leaf distances of gene tree branches, as an empirical measure of divergence from the clock; 0 corresponds to strict molecular clock and higher values correspond increased divergence (the x axis is in the log scale). Refer to Figs B and C in S1 Appendix for model conditions not shown here.

The new datasets, D1 and D2 are simulated using a similar approach. For each number of species in both D1 and D2, we simulated 10 different model conditions where we changed parameters that control divergence from the strict clock and the distance of the outgroup to the ingroups. Seven out of ten model conditions included an outgroup. The outgroup is added as a sister to the ingroups on the species tree. The length of the branch above ingroups (connecting them with the root) is decided by multiplying a fixed number by the height of the ingroup species tree; we refer to that fixed number as the root to crown ratio (R/C). For example, an R/C of 0.5 indicates that the branch connecting the root of the ingroups to the root of the tree is half the height of the ingroup tree. The choice of the R/C ratio directly impacts how often the species tree outgroup is also a gene tree outgroup (Fig 1C).

Beyond the R/C ratio, model conditions are also distinguished by two parameters of SimPhy that control deviations from the clock: (i) gene by lineage specific rate heterogeneity, which is a multiplier drawn from a gamma distribution for each branch of each gene tree, and (ii) species specific branch rate heterogeneity rate, which is also a multiplier drawn from a gamma distribution per species and is used to scale all gene tree branches for that species universally. The gamma distributions are mean-preserving, and therefore are specified with one shape parameter. We draw the value of that shape parameter from a log normal distribution with the scale hyperparameter *σ* = 1 and a varying location hyperparameter, which controls the level of deviation from the strict clock. We refer to the log normal location (which is the log of the mean of the distribution minus 0.5) as the clock deviation parameter; the higher values correspond to gamma distributions more closely centered around one, and thus, less deviation from the clock, while lower values correspond to more deviation (Fig 1D).

In six model conditions, the clock deviation parameter is fixed to a moderate value of 1.5, and the R/C ratio is varied between 0 (no outgroup), 0.25, 0.5, 1, 2, and 4. In the remaining model conditions, the R/C ratio is fixed to either 0 or 1 and the clock deviation parameter is changed between 0.15, 1.5, and 5 to get high, moderate, and low levels of deviations, respectively. Note that the two model conditions with heterogeneity hyperparameter 1.5 are common with six conditions that varied the R/C ratio; thus, in total we have ten model conditions for each number of species.

Other parameters of the SimPhy simulation procedure are sampled from distributions as described in Tables A and B in S1 Appendix. In D2, the expected species tree height in is set to 14.7 million generations, which is much higher than the 3 million used for the 30-taxon dataset. We chose different heights for small and large datasets because having 30 surviving species in a span of 3 million generations is reasonable, but having many thousands of extant species in such a short evolutionary time is unlikely. Thus, for the D2 dataset, we increased the height to obtain more realistic conditions.

The portion of quartet trees induced by gene trees that are found in the species tree can be used as a measure of ILS [68], where values close to 1/3 indicate extremely high levels of ILS and values close to 1 indicate no ILS. Our datasets varied between these two extremes (Fig 1A). The gene tree estimation error, measured by RF distance between true gene trees and estimated gene trees, was similarly heterogeneous and was also substantially impacted by deviations from the clock (Fig 1B); with low and medium deviations, median gene tree error was respectively 25% and 32%, while for high deviations, the error increased to 49%.

A major point of the current paper is that an outgroup species is not always an outgroup in gene trees, even in the *true* gene trees. When the R/C ratio is low, many of the true gene trees do not have the outgroup species in the outgroup position (Fig 1C). Interestingly, with the 30-taxon dataset, only at the extremely high R/C = 4 the outgroup is outside the ingroups in close to all gene trees of all replicate datasets. At the other extreme, with R/C = 0.25, in more than 50% of replicate runs, more than 50% of our 500 true gene trees did not have the outgroup species in the outgroup position. The larger datasets, which had higher numbers of generation and higher levels of ILS (Fig 1A) had fewer cases of outgroup mixing with ingroups in true gene trees.

#### Evaluation metrics

To estimate the accuracy of a rooted gene tree, we measure the proportion of all $\left(\begin{array}{c} n\\ 3 \end{array}\right)$ triplets in the reference (i.e., true) tree that are also found in the estimated tree. This measure is a function of both the accuracy of the unrooted topology of the estimated tree and the accuracy of the rooting. To separate the rooting error from the tree error, for the small 30-taxon dataset where it is feasible, we examine all possible root placements and find the “ideal” rooting that results in the lowest possible triplet distance (the ideal triplet distance is zero if and only if the unrooted tree is correct). We then define “delta triplet distance” as the difference between the triplet distance of the estimated tree with the rooting of interest (OG/MV/MP) and the triplet distance of the estimated tree with the ideal rooting. For the small trees with 30 leaves, we also afford to compute the rooted SPR distance using SPRDist [69]; however, for larger trees, SPR could not be computed. Finally, for the true unrooted gene trees that are rooted using an algorithm, we also report normalized branch distance, defined as the number of branches between the correct root and the estimated root, normalized by the maximum number of branches from any leaf to the root.

Beyond triplet distance, we use the normalized RF distance to measure the accuracy of unrooted trees, and we use percentage of quartets in the gene trees also present in the true gene trees (as computed by ASTRAL [48]) as a measure of ILS. For species trees, we also report the Matching Split measure [70]. We also report running time, measured on Intel EM64T Xeon nodes with 64GB memory.

#### Implementations

**Fig 2:**
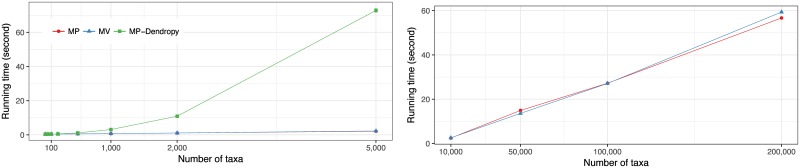
Running time of MP and MV. A: comparison of our implementation of MV/MP with the implementation of MP in Dendropy, which employs a quadratic algorithm, on datasets D1, D2, and D3 with up to 5,000 leaves; B: Linear time scaling of our implementation, tested on the RNASim dataset with up to 200,000 leaves.

We implemented both MP and MV (https://uym2.github.io/MinVar-Rooting/) using the Dendropy package for phylogenetic manipulations [71]. As expected, the running time of the algorithm increases linearly with the number of leaves (Fig 2); an RNASim [63] tree with 200,000 leaves could be rooted in just under a minute. In contrast, Dendropy seems to use a quadratic implementation of MP rooting (Fig 2A).

## Results

We will examine the following research questions using simulated and empirical data:

- *RQ1*: Does our novel MV rooting improve the root placement accuracy compared to MP and OG rooting for datasets with varying numbers of species?
- *RQ2*: How are MP, MV, and OG impacted by (i) gene tree estimation error, (ii), divergence from the clock, and (iii) outgroup distance to ingroups (R/C)?
- *RQ3*: What is the impact of rooting error on the species tree estimation, and is STAR less accurate than its unrooted counterpart, NJst?

### Simulation results

#### RQ1: MV for varying numbers of leaves

**Fig 3:**
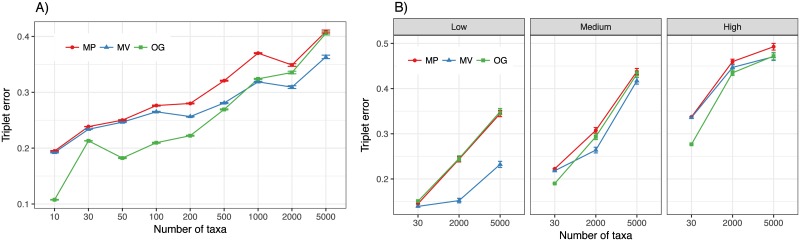
Absolute triplet distance as a function of the number of taxa. A: Results from D1, D2, and D3 are combined in one figure; 30 on the x-axis corresponds to D1, 2000 and 5000 to D2, and the remaining cases to D3. For D1 and D2, we fixed R/C = 1 and the clock divergence parameter to medium to best match the conditions of D3. B: Results for D1 and D2 with R/C = 1 and difference levels of clock divergence.

**Fig 4:**
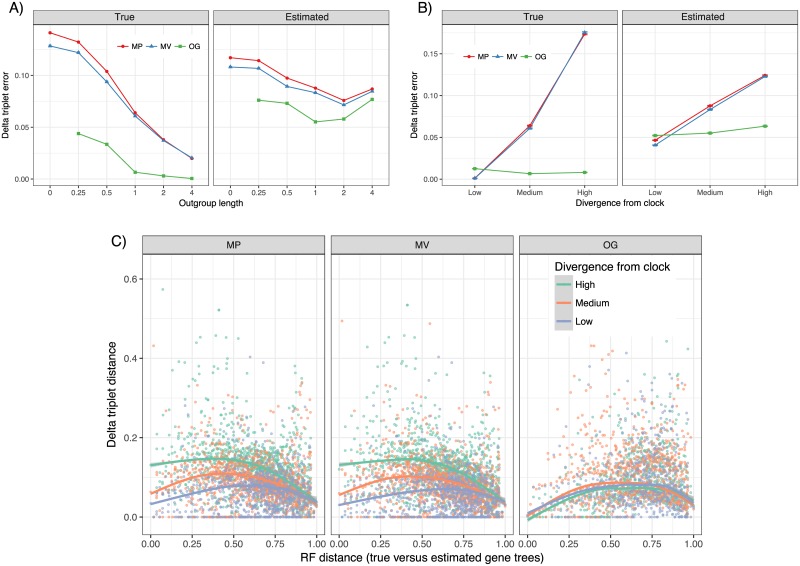
Rooting error above ideal rooting on 30-taxon dataset. Top: delta triplet error with both true and estimated gene trees for (A) medium divergence from the clock and varying R/C ratios and (B) R/C = 1 and varying levels of divergence from the clock. C: Delta triplet error versus gene tree estimation error, measured by RF distance, shown for high, medium, and low divergence from the clock; each point is an average of all gene trees in all replicates that had an identical RF gene tree error. A loess regression is fitted to the data using R.

On the D1 (30-taxon) dataset with estimated gene trees, MV matched or improved the triplet accuracy of MP in all 10 model conditions (Figs 3 and 4B, and Fig E in S1 Appendix). Overall, MV had lower error than MP (mean triplet error: 0.238 and 0.244, respectively), and the differences were statistically significant according to an analysis of variance (ANOVA) test comparing the two methods (*p* < 10^−5^), and considering divergence from the clock or the outgroup distance as other independent variables (to be discussed in RQ2). However, averaged over all 7 conditions of D1 where outgroups were available, OG rooting was more accurate than MV rooting, a pattern that was not universal and will require a nuanced consideration of parameter effects (RQ2).

When we combine D1, D2, and D3 to get a heterogeneous dataset that ranges between 10 to 5000 taxa, a clear pattern emerges. While with smaller numbers of species, OG performs the best, when the number of taxa is increased to 1000 and beyond, MV gradually becomes the most accurate method (Fig 3A). Increasing the number of taxa from 10 to 5,000 gradually increases the error for all methods, but OG is impacted more than MV (an increase from 0.1 triplet distance to 0.4 for OG, but from 0.2 to 0.35 for MV). MP is never the most accurate method but with trees of 5000 taxa, it is not worse than OG either. It is interesting to note that 2000 and 5000-taxon datasets, which have higher average tree height than 30-taxon datasets, have lower numbers of *true* gene trees where the outgroup species is not the gene tree outgroup (Fig 1C). Thus, the sharp decrease in the accuracy of OG is not related to increased impacts of ILS and has to be attributed to increased error in the estimated gene trees. When we focus on our new datasets (D1 and D2), it becomes clear that improvements of MV over OG are the most pronounced with lower deviations from the clock (Fig 3B).

#### RQ2: Impact of error, clock, and outgroup distance

We focus our discussion on D1, but patterns on the D2 dataset are similar Fig E in S1 Appendix. On D1, we focus on the triplet error, but SPR distance gives similar results Fig F in S1 Appendix.

While MV is always at least as good as MP in our simulations, on D1, improvements of MV compared to MP are significantly impacted by both the level of divergence from the clock and the R/C ratio (*p* = 0.002 and *p* < 10^−5^, respectively, according to the two-way ANOVA test). The improvements of MV over MP are higher when divergence from the clock is less and when the outgroup distance is smaller; the highest difference is for the case with no outgroup (Fig 4A, and Figs E-F in S1 Appendix).

The OG rooting is extremely accurate if true gene trees were known (Fig 4A and 4B, and Figs D and F in S1 Appendix); cases of error are limited to when the root is not very diverged from the ingroups (R/C< 1). In contrast, MV and MP, while are better than OG with low divergence from the clock, can have very high error rate even on true gene trees if divergences from the clock are sufficiently large (Fig 4B and Figs D and F in S1 Appendix). For example, MV (MP) finds a root that on average has 25% (30%) normalized branch distance to the correct root (Fig D in S1 Appendix); i.e., the inferred root is away from the correct root by a quarter of the maximum tree height.

On estimated gene trees, however, the accuracy of OG rooting severely degrades. The delta triplet error (triplet error above ideal rooting) of OG is only slightly better than MV with various R/C ratios with medium divergence from the clock (Fig 4A) and is worse than MV with low divergence from the clock (Fig 4B); OG remains substantially more accurate than MV with high divergence from the clock. Confirming this pattern, considering all individual genes in all replicates, as gene tree error increases from 0% to approx. 50%, delta triplet error seems to increase for all methods but the increase is more pronounced for OG (Fig 4C). Beyond 60% gene tree error (RF), delta triplet error actually goes down perhaps because even the ideal rooting has very high error, leaving little or no room for extra error due to rooting alone.

The delta triplet error of estimated gene trees rooted with OG reveals an interesting (U-shape) pattern. Choosing very small or very large R/C ratios (e.g., very close or distant outgroups) is not ideal (Fig 4A). Instead, the best performance is obtained by R/C = 1. This ratio seems to give outgroups that are as close as possible to the ingroups to reduce LBA effects while remaining sufficiently long to reduce impacts of ILS.

#### RQ3: Species tree error

We focus on the average RF distance here; using RF distributions (Fig G in S1 Appendix) or average distances according to the MS metric (Table C in S1 Appendix) do not change any of our conclusions.

The average RF error of species trees run on estimated gene trees with inferred roots ranges between 9.1% and 9.5% (Table 1). STAR run on the true gene trees with the true root has an average RF error of 5.8%; thus, a substantial part of the species tree error can simply be attributed to ILS and lack of insufficient number of gene trees to find a perfect species tree. STAR run on the gene trees with ideal rooting has 8.6% RF error, which is a 48% increase from STAR run on true gene trees. These differences are statistically significant according to a two-way ANOVA test where the clock divergence parameter is the second independent variable. Therefore, the second substantial contributor to the species tree error is the gene tree estimation error.

**Table 1:** Species tree estimation accuracy using rooted and unrooted gene trees.

Methods compared	p-value		Mean RF ST error	
	method	clock par.	1st method	2nd method
STAR True vs STAR Ideal	<10^−5^	0.126	0.058	0.086
STAR Ideal vs STAR OG	0.551	0.0009	0.086	0.091
STAR Ideal vs STAR MV	0.144	<10^−5^	0.086	0.095
STAR OG vs STAR MV	0.476	<10^−5^	0.091	0.095
STAR OG vs NJst	0.623	0.00005	0.091	0.093

ANOVA tests were performed on the D1 (30-taxon) dataset for pairs of methods. RF error is used as the metric. The tests were performed on the subset of D1 where outgroup exists. For true gene trees, the true root is known. For estimated gene trees, the Ideal is the rooting position that minimizes triplet error to the true gene trees. p-values are shown for the significance of differences between the error of the two methods specified in each row, and for the differences in error among the three levels of clock divergence parameter, respectively.

Despite all the differences observed in the accuracy of rooting individual gene trees, we surprisingly found no clear evidence that the rooting error has a significant impact on the species tree accuracy. The RF error of STAR species trees run on estimated gene trees with ideal rooting (which uses the known true gene tree) was not significantly different from that of the STAR run on estimated gene trees rooted using OG or MV (Table 1). We also saw no statistically significant differences between species trees estimated from gene trees rooted using MV or OG. Thus, given estimated gene trees, which in our dataset had high rates of error (Fig 1B), the delta error due to rooting inaccuracies does not seem to lead to much further reduction in accuracy. Consistent with this hypothesis, we also observed no statistically significant differences (Table 1) between STAR rooted using OG and NJst (which due to its strong parallels with STAR can be called unrooted STAR [52]).

On estimated gene trees, all rooting methods are negatively impacted by increased deviations from a strict clock (Table 1 and Table C in S1 Appendix). The reduction may relate to increased unrooted gene tree estimation error with increased deviations (Fig 1B); it may also be related to the fact that rooting becomes successively harder with stronger deviations from the strict clock (Fig 4B).

### Biological results

We tested MV rooting on an angiosperm dataset with 46 species and 310 genes [72], where the correct rooting has been a point of debate [42]. This dataset includes a single outgroup (*Selaginella*). We rooted each gene tree using both OG and MV, and compared gene trees with the published MP-EST species tree [49, 72] using the triplet distance *after* removing the outgroup from the gene trees and the species tree. The motivation for using this score is that we conjecture an incorrect rooting will tend to increase observed discordance of gene trees with the species tree. On this dataset, OG and MV essentially result in the same average triplet distance to the MP-EST species tree (19.2% for OG and 19.3% for MV) and their differences are not statistically significant (p-value = 0.9). It’s worth noting that excluding outgroups could have had reduced gene tree estimation error, and therefore, may have been a better approach overall.

## Discussion

Our simulations made it clear that even if the outgroup distance to ingroups is twice as much as the most distant ingroups (i.e., R/C = 1), there can still be many *true* gene trees that fail to have the outgroup as sister to the ingroups (Fig 1C). How often such cases of outgroup/ingroup mixing happens depends on the level of ILS, and by extension on the depth of the species tree and population size. Our 30-taxon dataset had numbers of generations that ranged between 407*K* and 9.1*M* generations in 90% of replicates; thus, our trees range between relatively shallow to moderately deep. Overall congruence of gene trees with the species tree, as measured by the quartet score, was high (> 0.8) for 43% of replicates, and was moderate (0.6 – 0.8) for another 34%. Thus, despite having realistic conditions, we observe outgroups mixed with ingroups in true gene trees.

Making outgroups maximally distant from ingroups, however, won’t solve the problem. As Rosenfeld *et al*. have pointed out [22], making the outgroups distant can lead to random assignment of outgroups in the gene trees, thereby increasing the apparent discordance. In agreement with their results, and much of the literature, we found that very distant outgroups, while placed as desired in the true gene trees, can lead to increased overall error (Fig 4A). There is a trade-off between making the outgroup closer to ingroups to minimize LBA and making it more distant to reduce ILS; in our 30-taxon dataset and under our conditions of simulations, the optimal setting was R/C = 1, corresponding to outgroups that are twice as distant from any of the ingroups as the most two divergent ingroups. The exact optimal value, however, likely depends on the exact parameters of a biological dataset and the choice of R/C = 1 cannot be blindly prescribed.

Increased divergence from the clock substantially increased unrooted gene tree estimation error (Fig 1B), but impacted the accuracy of rooting only when MP or MV were used (Fig 4B). The strong dependence of gene tree estimation on clock assumptions leads us to suggest that simulations of the MSC process should always include conditions where the strict clock are violated. Many methods are proved consistent and tested empirically only under the strict clock assumption, a situation that we hope our results will change. New simulation tools such as SimPhy make it easy to simulate datasets that deviate from the strict clock assumption.

A surprising result of our simulation studies was that while gene tree rooting error was generally high, we could not detect a significant impact on the species tree. Two explanations have to be considered. It could be that in general the impact of rooting error on species tree estimation is minimal. On the other hand, the lack of power to detect significant impact may be limited to specifics of our simulation procedure. Several important parameters of the simulation may have reduced the effect of rooting error on species tree estimation error. We always had five hundred genes, which is relatively high considering that we only had 30 ingroup species. Impact of rooting error for datasets with more species and/or fewer genes may be different, a problem that we did not get to address here because of computational limitations. Moreover, we conjecture that at least part of the reason for this lack of observed impact is that our datasets had high levels of gene tree estimation error even for the unrooted tree. It is conceivable that the impact from mis-rooting is drawn out by the impact of topological error and is hard to detect with a datasets of 100 replicates, simulated with heterogeneous parameters drawn from wide parameter distributions. We note that our simulation setup was designed mainly to address the question of gene tree rooting error and to enable a comparison between our new MV and existing MP and OG rooting methods. Moreover, we focused only on comparing NJst and STAR because of their deep mathematical connection; our current study cannot be generalized to other methods such as ASTRAL and MP-EST (which can in principle be altered to take as input both rooted and unrooted trees). Thus, while our results are suggestive that there may be considerable robustness to gene tree rooting error at least among some methods, to arrive at a more nuanced understanding of impacts of rooting, simulation setups designed directly to answer this questions will be needed in future.

Several other limitations of our study should be noted. In our simulations, we always included only one outgroup (a limitation of SimPhy), but the impact of selecting multiple outgroups will be important to examine. We inferred gene trees under the exact model of sequence evolution that generated the data, but the impact of factors such as LBA are known to be exacerbated by model misspecification. Our deviations from the clock were random and did not depend on time. Finally, more realistic models of change in evolutionary tempo may result in more systematic biases and different conclusions.

## Conclusion

We introduce a new method for rooting phylogenetic trees, which relies on minimizing the variance of the root to tip distances. The method can be efficiently implemented in an algorithm that scales linearly with increased number of species and runs in less than a minute for datasets of up to 200,000 leaves. Our new approach is more accurate than the traditional midpoint rooting and its relative accuracy compared to the dominant method of outgroup rooting depends on the number of species; with very large trees, minimizing root to tip variance outperforms outgroup rooting whereas for small and moderate size datasets outgroups are more accurate. Regardless of the relative accuracy of methods, we showed that rooting gene trees is challenging because deviations from a strict clock make it hard for automatic methods to find the correct root, while gene tree discordance makes outgroup rooting unreliable. However, within the limitations of our study, we detected no significant impact due to gene tree error on the accuracy of the species tree accuracy for datasets with large numbers of gene trees, many of them inferred from datasets with low phylogenetic signal. We leave a more nuanced consideration of impacts of incorrect rooting on species tree error to future research.

## Supporting information

S1 Appendix (PDF)
