# Supplementary material for: Minimum variance rooting of phylogenetic trees and implications for species tree reconstruction
Source: PLoS One. 2017 Aug 11;12(8):e0182238. doi: 10.1371/journal.pone.0182238 (PMC5553649; doi:10.1371/journal.pone.0182238)
Supplement: S1 Appendix — (PDF) [file pone.0182238.s001.pdf]

Supplementary Theory

In this section we prove the following propositions, which were used in the main text to support the theory of the MinVar rooting method. Please refer to the main paper for more details.

Proposition 1. *A point p on tree T is a local MV if and only if it is a balance point.*

Based on Proposition 1, we refer to local MV and balance point interchangeably.

Proposition 2. *Any tree has at least one local MV.*

Proposition 3. *The global MV of any tree is one of its local MVs.*

Proposition 4. *Let p denote the global MV of T. If*

$$\epsilon \leq \min_{w \in c(r)} \left(\frac{e_w}{\frac{n}{n-|w|}h + e_w} \right)$$

then there exists a child w of r such that $p \in e(r, w)$

Proposition 5. *When the global MV is on one of the adjacent edges of r, let a random variable X indicate the distance of the global MV to the root; then, $E(X) = 0$.*

Proposition 6. *Let p be a point on an edge (u, v) of tree T with distance $d(p, u) = x$. If we let p vary along edge (u, v) and consider $var(p)$ as a function of variable x with parameters u and v, then:*

$$var(p) = var(x; u, v) = (1 - \beta^2)x^2 + \left(\alpha - \frac{2ST(u)\beta}{n}\right)x + var(u) \tag{S1}$$

in which

$$\alpha = \frac{2ST(u) - 4(SI(v) + |v|e_v)}{n} \quad \text{and} \quad \beta = 1 - \frac{2|v|}{n} \tag{S2}$$

Extra notations

For two points p and p', potentially on different edges, we let $path(p, p')$ denote the *directed* path from p to p'. For two nodes p and u, we define $Cld_p(u)$ as the clade under u if the tree T is rerooted at p. For ease of notation we use $|p \triangleright u|$ to denote the size of $Cld_p(u)$. For a point p on tree T and another point p' on either the same edge or an edge connected to p (if p is a node), we let $\overrightarrow{pp'}$ denote a *direction* of p. It is easy to see that any point on a tree has at least two directions, and any node that is not the root has at least three directions. We call $\overrightarrow{pp'}$ a *dominant* direction of p if and only if

$$\frac{1}{|p \triangleright p'|} \sum_{i \in Cld_p(p')} d_i(p) > \frac{1}{n - |p \triangleright p'|} \sum_{i \notin Cld_p(p')} d_i(p) \tag{S3}$$

Proofs

Proofs of ST relation and Proposition 6

On a tree T, consider a point p on the edge (u, v) with distance x from u (Fig A).

Proof of ST relation. Recall that $ST(v)$ is the sum of distances of all leaves from the node v (i.e. $ST(p) = \sum_{i \in Cld(p)}(d_i(p))$. We need to prove that

$$ST(v) = ST(p(v)) + (n - 2|v|)e_v. \tag{S4}$$

We have

$$\begin{aligned} ST(p) &= \sum_{i \in Cld(p)} (d_i(u) - x) + \sum_{i \in L - Cld(p)} (d_i(u) + x) \\ &= \sum_{i \in L} d_i(u) + (|L| - |p| - |p|)x \\ &= ST(u) + (n - 2|p|)x \end{aligned} \tag{S5}$$

Let $p \equiv v$, we get Eq. S4. □

Proof of Proposition 6. Recall that $ST(p) = \sum_{i \in L} d_i(p)$.

$$var(p) = \frac{1}{n} \sum_{i \in L} (d_i(p) - \frac{\sum_{i \in L} d_i(p)}{n})^2 = \frac{\sum_{i \in L} d_i^2(p)}{n} - (\frac{ST(p)}{n})^2 \tag{S6}$$

The first term of the RHS of S6 can be expanded as follow:

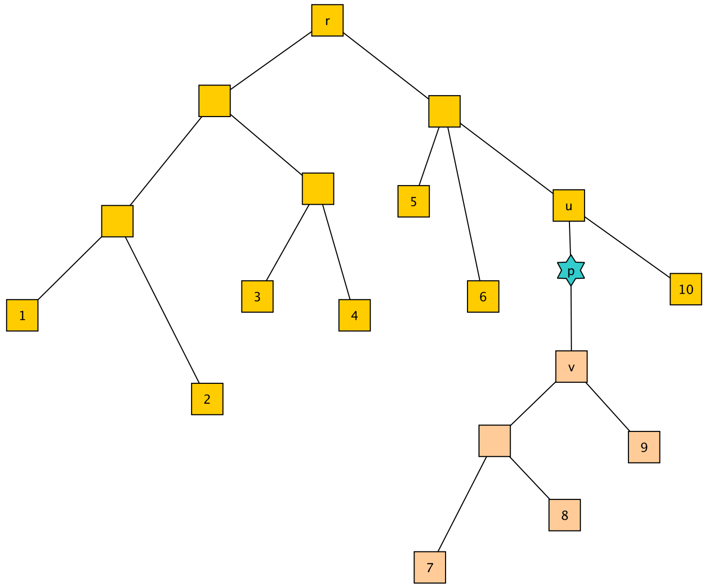

Fig A. An example tree T rooted at r with a point p on edge (u, v).

$$
\begin{aligned}
\frac{\sum_{i\in L} d_i^2(p)}{n} &= \frac{1}{n}\sum_{i\in Cld(v)}(d_i(u)-x)^2 + \frac{1}{n}\sum_{i\in L-Cld(v)}(d_i(u)+x)^2 \\
&= \frac{1}{n}\sum_{i\in Cld(v)}(d_i^2(u)-2d_i(u)x+x^2) + \frac{1}{n}\sum_{i\in L-Cld(v)}(d_i^2(u)+2d_i(u)x+x^2) \\
&= \frac{1}{n}\sum_{i\in L} d_i^2(u) + \frac{2}{n}(\sum_{i\in L-Cld(v)} d_i(u) - \sum_{i\in Cld(v)} d_i(u))x + x^2 \\
&= \frac{1}{n}\sum_{i\in L} d_i^2(u) + 2x\frac{\sum_{i\in L} d_i(u) - 2\sum_{i\in Cld(v)} d_i(u)}{n} + x^2 \\
&= \frac{1}{n}\sum_{i\in L} d_i^2(u) + 2x\frac{ST(u) - 2\sum_{i\in Cld(v)}(d_i(v)+e_v)}{n} + x^2 \\
&= \frac{1}{n}\sum_{i\in L} d_i^2(u) + 2x(\frac{ST(u) - 2(SI(v)+|v|e_v)}{n}) + x^2 \\
&= \frac{1}{n}\sum_{i\in L} d_i^2(u) + \alpha x + x^2
\end{aligned} \tag{S7}
$$

where the last line is simply derived from the definition:

$$\alpha = \frac{2ST(u) - 4(SI(v)+|v|e_v)}{n}$$

Recall $\beta = (1 - \frac{2|v|}{n})$; the second term can be expanded as follow:

$$
\begin{aligned}
\left(\frac{ST(p)}{n}\right)^2 &= \left(\frac{ST(u)+(n-2|v|)x}{n}\right)^2 \\
&= \left(\frac{ST(u)}{n} + \beta x\right)^2 \\
&= \left(\frac{ST(u)}{n}\right)^2 + \frac{2ST(u)\beta x}{n} + \beta^2 x^2
\end{aligned} \tag{S8}
$$

Substitute S7 and S8 to S6, we obtain:

$$
\begin{aligned}
var(p) &= \frac{\sum_{i\in L} d_i^2(u)}{n} + \alpha x + x^2 - \left(\frac{ST(u)}{n}\right)^2 - \frac{2ST(u)\beta x}{n} - \beta^2 x^2 \\
&= \frac{\sum_{i\in L} d_i^2(u)}{n} - \left(\frac{ST(u)}{n}\right)^2 + \left(\alpha - \frac{2ST(u)\beta}{n}\right)x + (1-\beta^2)x^2 \\
&= var(u) + \left(\alpha - \frac{2ST(u)\beta}{n}\right)x + (1-\beta^2)x^2
\end{aligned} \tag{S9}
$$

Thus, we get Eq. S1 □

Useful Lemmas

Below are useful lemmas that will be used later in the proofs.

Lemma 1. *Any point on a tree either is a balance point or has at least one dominant direction.*

Proof. On tree T, consider an arbitrary point p that is adjacent to nodes $v_1, v_2, ..., v_k$ of T. Let $\mu_j = \frac{1}{|p \triangleright v_j|} \sum_{i \in Cld_p(v_j)} d_i(p)$. If $\mu_1 = \mu_2 = ... = \mu_k$, then p is a balance point of T. Otherwise, let $\mu_m = max(\mu_1, \mu_2, ..., \mu_k)$. It is easy to see that $\overrightarrow{pv_m}$ is a dominant direction of p. □

Lemma 2. *If a point p_0 is not a local MV of tree T, there exists at least one point p' on T such that $var(p') < var(p_0)$.*

Lemma 3. *Consider an edge $e = (u, v)$ of tree T. If $\overrightarrow{uv}$ is a dominant direction of u and $\overrightarrow{vu}$ is a dominant direction of v, then there exists a balance point on edge e.*

(Lemmas 2 and 3 are proved later)

Proofs of Proposition 1 and Lemma 2

We start by some definitions and derivations that are used in proofs of both Proposition 1 and Lemma 2. Consider a point p_0 on tree T and any arbitrary point p on the same edge as p_0 or on an edge adjacent to p_0 if p_0 is a node. Note that when p_0 is in the middle of a edge, p can be a point above or below it on the same edge, but when p_0 is a node, p can be a point on any of the three (or more) edges adjacent to p. We divide the leaf set L of T into two disjoint groups: the leaves inside $Cld_{p_0}(p)$ (group 1), and the remaining leaves (group 2). Let $x = d(p_0, p)$, n be the size of T, and k be the size of group 1; the size of group 2 is therefore $n - k$. Let $d'_1, d'_2, ..., d'_k$ be the distances of the leaves in group 1 to p_0, $d'_{k+1}, d'_{k+2}, ..., d'_n$ be the distances of the leaves in group 2 to p_0, $d_1, d_2, ..., d_k$ be the distances of the leaves in group 1 to p, and $d_{k+1}, d_{k+2}, ..., d_n$ be the distances of the leaves in group 2 to p. Also let μ' and μ be the averages of the leaf distances to p_0 and p. Then:

$$d_i = \begin{cases} d'_i - x, & \text{if } 1 \le i \le k \\ d'_i + x, & \text{if } k+1 \le i \le n \end{cases} \tag{S10}$$

$$\mu' = \frac{1}{n}\left(\sum_{i=1}^{n} d'_i\right) \qquad var(p_0) = \frac{\sum_{i=1}^{n}(d'_i)^2}{n} - \mu'^2 \tag{S11}$$

$$\mu = \frac{1}{n}\sum_{i=1}^{n} d_i = \frac{1}{n}\left(\sum_{i=1}^{n} d'_i\right) + \frac{n-2k}{n}x = \mu' + \frac{n-2k}{n}x \tag{S12}$$

$$\begin{aligned} var(p) = \frac{\sum_{i=1}^{n} d_i^2}{n} - \mu^2 &= \frac{1}{n}\left(\sum_{i=1}^{k}(d'_i - x)^2 + \sum_{i=k+1}^{n}(d'_i + x)^2\right) - \left(\mu' + \frac{n-2k}{n}x\right)^2 \\ &= var(p_0) + \left(1 - (\frac{n-2k}{n})^2\right)x^2 + \frac{2}{n}x\left((\sum_{i=k+1}^{n} d'_i) - (\sum_{i=1}^{k} d'_i) - (n-2k)\mu'\right) \end{aligned} \tag{S13}$$

$$\frac{var(p) - var(p_0)}{x} = \left(1 - (\frac{n-2k}{n})^2\right)x + \frac{2}{n}\left((\sum_{i=k+1}^{n} d'_i) - (\sum_{i=1}^{k} d'_i) - (n-2k)\mu'\right) \tag{S14}$$

Let $x \to 0$, we have

$$lim_{x\to 0}\frac{var(p)-var(p_0)}{x} = \frac{2}{n}\left((\sum_{i=k+1}^{n} d'_i) - (\sum_{i=1}^{k} d'_i) - (n-2k)\mu' \right) \quad \text{(S15)}$$

Proof of Proposition 1. We consider both directions.

a. Suppose p_0 is a local MV of T then by Eq. S15

$$\begin{aligned} &(\sum_{i=k+1}^{n} d'_i) - (\sum_{i=1}^{k} d'_i) - (n-2k)\mu' = 0 \\ \implies & n\sum_{i=k+1}^{n} d'_i - n\sum_{i=1}^{k} d'_i - (n-2k)\sum_{i=1}^{n} d'_i = 0 \\ \implies & \frac{1}{k}\sum_{i=1}^{k} d'_i = \frac{1}{n-k}\sum_{i=k+1}^{n} d'_i \end{aligned} \quad \text{(S16)}$$

Thus, p_0 is also a balance point, which completes one direction of Proposition 1.

b. Suppose p_0 is a balance point of T; then,

$$\frac{1}{k}\sum_{i=1}^{k} d'_i = \frac{1}{n-k}\sum_{i=k+1}^{n} d'_i = \mu' \quad \text{(S17)}$$

Substituting $\sum_{i=k+1}^{n} d'_i$ and $\sum_{i=1}^{k} d'_i$ in Eq. S15 gives

$$lim_{x\to 0}\frac{var(p)-var(p_0)}{x} = ((n-k)-k-(n-2k))\mu' = 0 \quad \text{(S18)}$$

which means, p_0 is a local MV. This completes the proof for Proposition 1. □

Proof of Lemma 2. Suppose p_0 is not a local MV. By Lemma 1, there is a point p_1 on the same edge or an adjacent edge to p_0 such that $\overrightarrow{p_0p_1}$ is a dominant direction of p_0. Letting $y = d(p_0, p_1)$, replacing p with p_1 in Eq. S15, we get:

$$\begin{aligned} lim_{y\to 0}\frac{var(p_1)-var(p_0)}{y} &= \\ \frac{2}{n^2}\big(n\sum_{i\notin Cld_{p_0}(p_1)} d_i(p_0) - n\sum_{i\in Cld_{p_0}(p_1)} d_i(p_0) - (n-2|p_0 \triangleright p_1|)\sum_{i\in L} d_i(p_0)\big) &= \\ \frac{4}{n^2}\big(|p_0 \triangleright p_1|\sum_{i\notin Cld_{p_0}(p_1)} d_i(p_0) - (n-|p_0 \triangleright p_1|)\sum_{i\in Cld_{p_0}(p_1)} d_i(p_0)\big) &< 0 \end{aligned}$$

where the inequality follows from the fact that $\overrightarrow{p_0p_1}$ is a dominant direction (see Eq. S3). Because the derivative at p_0 approaching from p_1 is negative, there exist a point p' in a small local neighborhood of p_0 towards p_1 such that $var(p') < var(p_0)$. □

Proofs of Proposition 2 – 5 and Lemma 3

Proof of Lemma 3. For the the edge (u, v) (where $u = p(v)$), let $m_1^u = \frac{1}{|u \triangleright v|}\sum_{i\in Cld_u(v)} d_i(u)$ and $m_2^u = \frac{1}{n-|u\triangleright v|}\sum_{i\notin Cld_u(v)} d_i(u)$, and similarly, $m_1^v = \frac{1}{|v\triangleright u|}\sum_{i\notin Cld_v(u)} d_i(v)$ and $m_2^v = \frac{1}{n-|v\triangleright u|}\sum_{i\in Cld_v(u)} d_i(v)$.

By definition of dominant direction (Eq. S3), $m_1^u > m_2^u$ and $m_1^v > m_2^v$. On the other hand, since $m_1^u = m_2^v + e_v$ and $m_2^u = m_1^v - e_v$, we have $0 < m_1^u - m_2^u = m_2^v - m_1^v + 2e_v < 2e_v$. Let p be a point on edge e such that $d(p,u) = x = \frac{m_1^u - m_2^u}{2}$. We have:

$$\frac{1}{|p \triangleright u|}\sum_{i \in Cld_p(u)} d_i(p) = m_1^u - x \quad \text{and} \quad \frac{1}{n - |p \triangleright u|}\sum_{i \notin Cld_p(u)} d_i(p) = m_2^u + x$$

$\frac{1}{|p \triangleright u|}\sum_{i \in Cld_p(u)} d_i(p) - \frac{1}{n-|p \triangleright u|}\sum_{i \notin Cld_p(u)} d_i(p) = m_1^u - m_2^u - 2x = 0$. Thus, p is a balance point of T. □

Proof of Proposition 2. Consider a tree T rooted at r_T. If r_T is a local MV, then the proof is complete. If r_T is not a local MV, by Lemma 1 and Lemma 3, there exists an edge $e_0 = (r_T, v_0)$ such that $\overrightarrow{r_T v_0}$ is a dominant direction of r_T. If v_0 is a balance point of T, or $\overrightarrow{v_0 r_T}$ is a dominant direction of v_0, then by Lemma 3 and Proposition 1, there is a local MV p on e_0.

Otherwise, by Lemma 1, v_0 has a dominant direction $\overrightarrow{v_0 v_1}$ associated with edge $e_1 = (v_0, v_1)$. Similar to the previous case, if v_1 is a balance point or $\overrightarrow{v_1 v_0}$ is a dominant direction of v_1, then there is a balance point p on e_1. Otherwise, v_1 has a dominant direction $\overrightarrow{v_1 v_2}$ associated with edge $e_2 = (v_1, v_2)$.

The process can be continued until we reach an edge $e_k = (v_{k-1}, v_k)$ such that either there is a local MV $p \in e_k$ or v_k is a leaf of T. If v_k is a leaf, then it is obvious that $\overrightarrow{v_k v_{k-1}}$ is a dominant direction of v_k. Recall that $\overrightarrow{v_{k-1} v_k}$ is a dominant direction of v_{k-1}. By Lemma 3 and Proposition 1, there is a local MV point p on e_k.

Thus, we can always find at least one local MV in a tree T (if tree T is finite). This completes the proof of Proposition 2.

□

Proof of Proposition 3. (Proof by contradiction) Suppose there exists a tree T with a global MV p_0 that is not a local MV. Let edge $e = (u, v)$ be the edge that contains p_0. Since p_0 is not a local MV, by Lemma 2, there exists a point p such that $var(p) < var(p_0)$, which contradicts the definition of global MV. □

Proof of Proposition 4. On tree T, let p be the global MV and $x = d(p, r)$, w denote the child of r that is on the same side as p, and d_i be the shorthand for $d_i(r)$ (i.e. the distance from r to leaf i *of tree* T). We prove that $x \leq (1-\epsilon)e_w$, and therefore, $p \in e(r_0, w)$. Note that T_0 and T have the same topology but are different in branch lengths. In this proof we use e_v to denote the length of the edge $(p(v), v)$ *of* T_0.

Follow the lemma condition

$$\epsilon \leq \frac{e_w}{\frac{n}{n-|w|}h + e_w} \implies \frac{n}{n-|w|}\epsilon h \leq (1-\epsilon)e_w \tag{S19}$$

By Proposition 1 and 3, p is a balance point. Therefore,

$$\frac{1}{|p|}\sum_{i \in Cld(p)} (d_i - x) = \frac{1}{|p|}\sum_{i \in Cld(p)} d_i(p) = \frac{1}{n-|p|}\sum_{i \notin Cld(p)} d_i(p) \tag{S20}$$

Also,

$$\frac{1}{n-|p|}\sum_{i \notin Cld(p)} d_i(p) \geq \frac{1}{n-|p|}\Big(\sum_{i \notin Cld(p)} (d_i) + (n-|w|)x - (|w|-|p|)x\Big) \tag{S21}$$

From Eq. S20 and S21, we have

$$\begin{aligned}
&\frac{\sum_{i\in Cld(p)}(d_i - x)}{|p|} \geq \frac{\sum_{i\notin Cld(p)} d_i + (n-|w|)x - (|w|-|p|)x}{n-|p|} \\
\implies &\frac{\sum_{i\in Cld(p)} d_i}{|p|} - x \geq \frac{\sum_{i\notin Cld(p)} d_i}{n-|p|} + \frac{(n-|w|)-|w|+|p|}{n-|p|}x \\
\implies &\left(1 + \frac{n-|w|-|w|+|p|}{n-|p|}\right)x = \frac{2(n-|w|)}{n-|p|}x \leq \frac{\sum_{i\in Cld(p)} d_i}{|p|} - \frac{\sum_{i\notin Cld(p)} d_i}{n-|p|}
\end{aligned}$$

Recall that under our model, T_0 is an ultrametric tree, so that for each leaf i, $\sum_{v\in path(i,r)} e_v = h$. Also, T was obtained by multiplying each edge of T_0 by a random variable with support $[1-\epsilon, 1+\epsilon]$. Thus, $(1-\epsilon)h \leq d_i = \sum_{v\in path(i,r)} e_v\alpha_v \leq (1+\epsilon)h$. Therefore,

$$\frac{2(n-|w|)}{n}x \leq \frac{2(n-|w|)}{n-|p|}x \leq 2\epsilon h \implies x \leq \frac{n}{n-|w|}\epsilon h \leq (1-\epsilon)e_w$$

Hence, there exists a child w of r such that the global MV belongs to edge (r, w). □

Proof of Proposition 5. Let D_i be the random variable corresponding to the distribution of $d_i(r)$ and P be a random variable giving the position of the global MV root. Then,

$$\begin{aligned}
E[D_i] &= E[\sum_{v\in path(i,r)} e_v\alpha_v] = \sum_{v\in path(i,r)} E[e_v\alpha_v] \\
&= \sum_{v\in path(i,r)} e_v E[\alpha_v] = \sum_{v\in path(i,r)} e_v = h
\end{aligned} \tag{S22}$$

By the global balance property of P, we can compute

$$X = \frac{1}{2}\left(\frac{\sum_{i\in Cld(P)} D_i}{|P|} - \frac{\sum_{i\notin Cld(P)} D_i}{n-|P|}\right) \tag{S23}$$

and thus,

$$E[X] = \frac{1}{2}\left(\frac{\sum_{i\in Cld(P)} E[D_i]}{|P|} - \frac{\sum_{i\notin Cld(P)} E[D_i]}{n-|P|}\right) = \frac{1}{2}(h-h) = 0 \tag{S24}$$

□

Supplementary Figures and tables

Table A. Parameters used in SimPhy simulation

Arg.	Description	Value for D1	Value for D2
RS	Number of replicates	100	20
RL	Number of loci	500	50
RG	Number of genes	1	
SB	Speciation rate	Log normal(1.0e-7,1.0e-6)	
SD	Extinction rate	Log normal(1.0e-7,SB)	
ST	Maximum tree length	Log normal(14.41412,1)	Log normal(16,1)
SL	Number of taxa	30	
SO	Root to crown ratio	R/C	
SI	Number of individuals per species	1	
SP	Global population size	Uniform(10000,1000000)	
SU	Global substitution rate	Log normal(-17.27461,0.6931472)	
HH	Gene by lineage specific locus tree parameter	1	
HS	Species specific branch rate heterogeneity rates	Log normal(α,1)	
HL	Gene family specific rate heterogeneity rates	Log normal(1.551533,0.6931472)	
HG	Gene by lineage specific rate heterogeneity rates	Log normal(α,1)	
CS	Random number generator seed	9644	

Root to crown ratios and Divergence from the strict clock are shown with variables α and R/C. These parameters change for each model condition and are available in Table B.

Table B. R/C **and** α **for different model conditions in datasets D1 and D2.**

Model Condition.	R/C for D1 and D2	α for D1 and D2
1	0	1.5
2	0.25	1.5
3	0.5	1.5
4	1	1.5
5	2	1.5
6	4	1.5
7	1	0.15
8	1	5
9	0	0.15
10	0	5

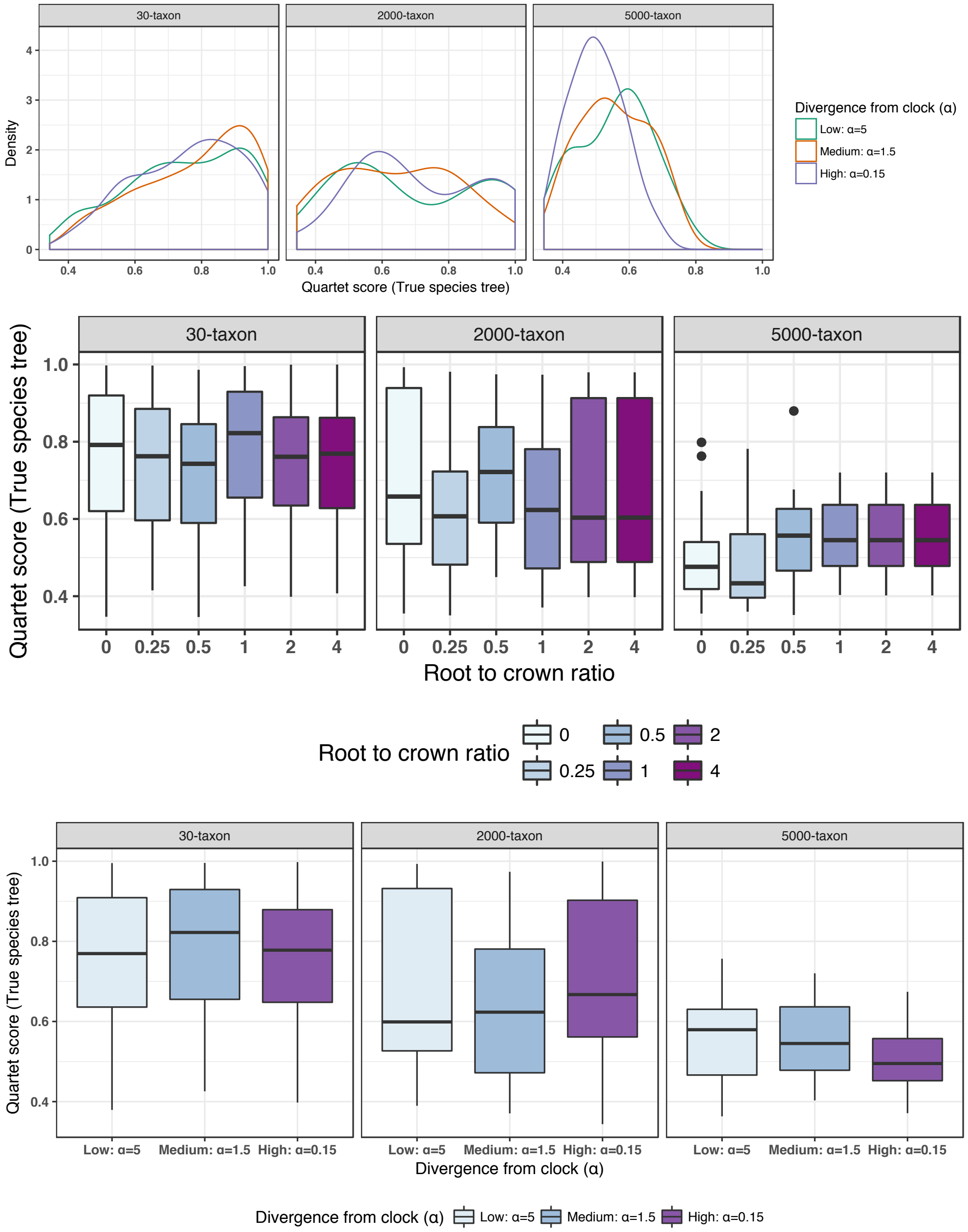

Fig B. ILS levels for new simulated datasets D1 and D2. Density plots (top) and box plots (middle and bottom) are shown for the quartet score of the true species tree with respect to the true gene trees, as a measure of the amount of ILS. Top: R/C=1. Middle: divergence from the clock = 1.5. Bottom: R/C=1.

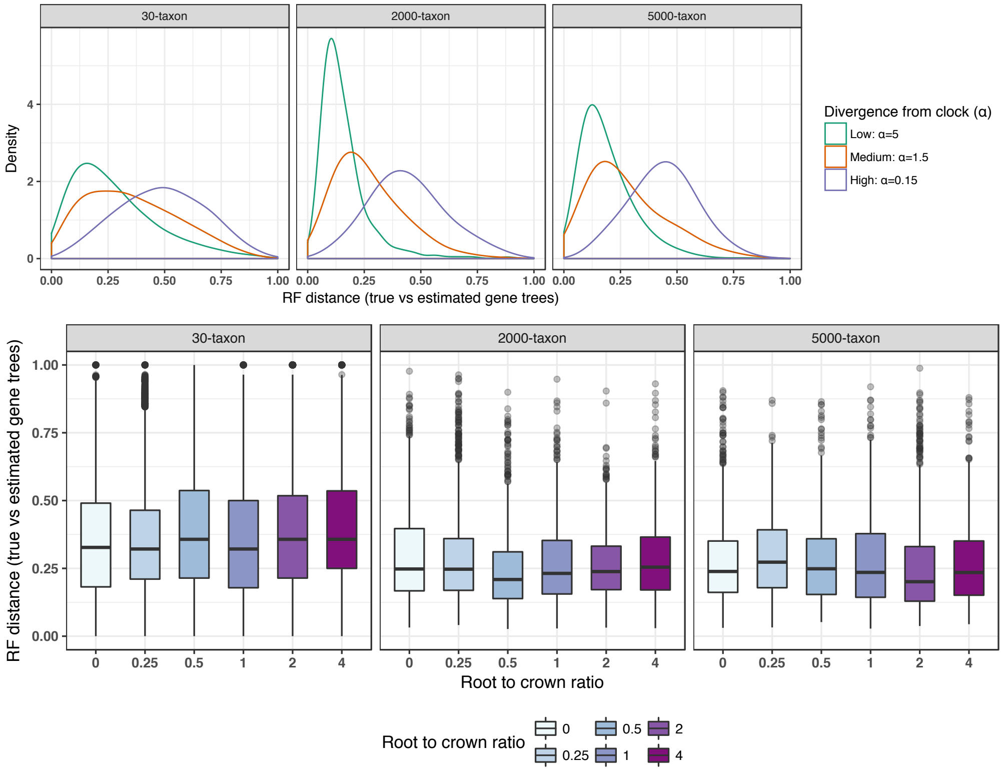

Fig C. Gene tree estimation error for datasets D1 and D2. The normalized RF distance is shown between true gene trees and the estimated gene trees. Top: density plots with R/C= 1; Bottom: boxplots with the divergence clock parameter set to 1.5.

Table C. Species tree estimation accuracy using rooted and unrooted gene trees

Methods compared	p-value		Mean MS ST error	
	method	clock par.	1st method	2nd method
STAR True vs STAR Ideal	$< 10^{-5}$	0.0638	7.6313	7.6313
STAR Ideal vs STAR OG	0.5820	0.0041	11.8875	12.0844
STAR Ideal vs STAR MV	0.1892	0.0008	11.8875	13.0938
STAR OG vs STAR MV	0.4768	0.0008	12.0844	13.0938
STAR OG vs NJst	0.1619	0.0085	12.0844	13.5906

ANOVA tests were performed on the D1 (30-taxon) dataset for pairs of methods. Matching-split (MS) error is used as the metric. The tests were performed on the subset of D1 where outgroup exists. For true gene trees, the true root is known. For estimated gene trees, the Ideal is the rooting position that minimizes triplet error to the true gene trees. p-values are shown for the significance of differences between the error of the two methods specified in each row, and for the differences in error among the three levels of clock divergence parameter, respectively.

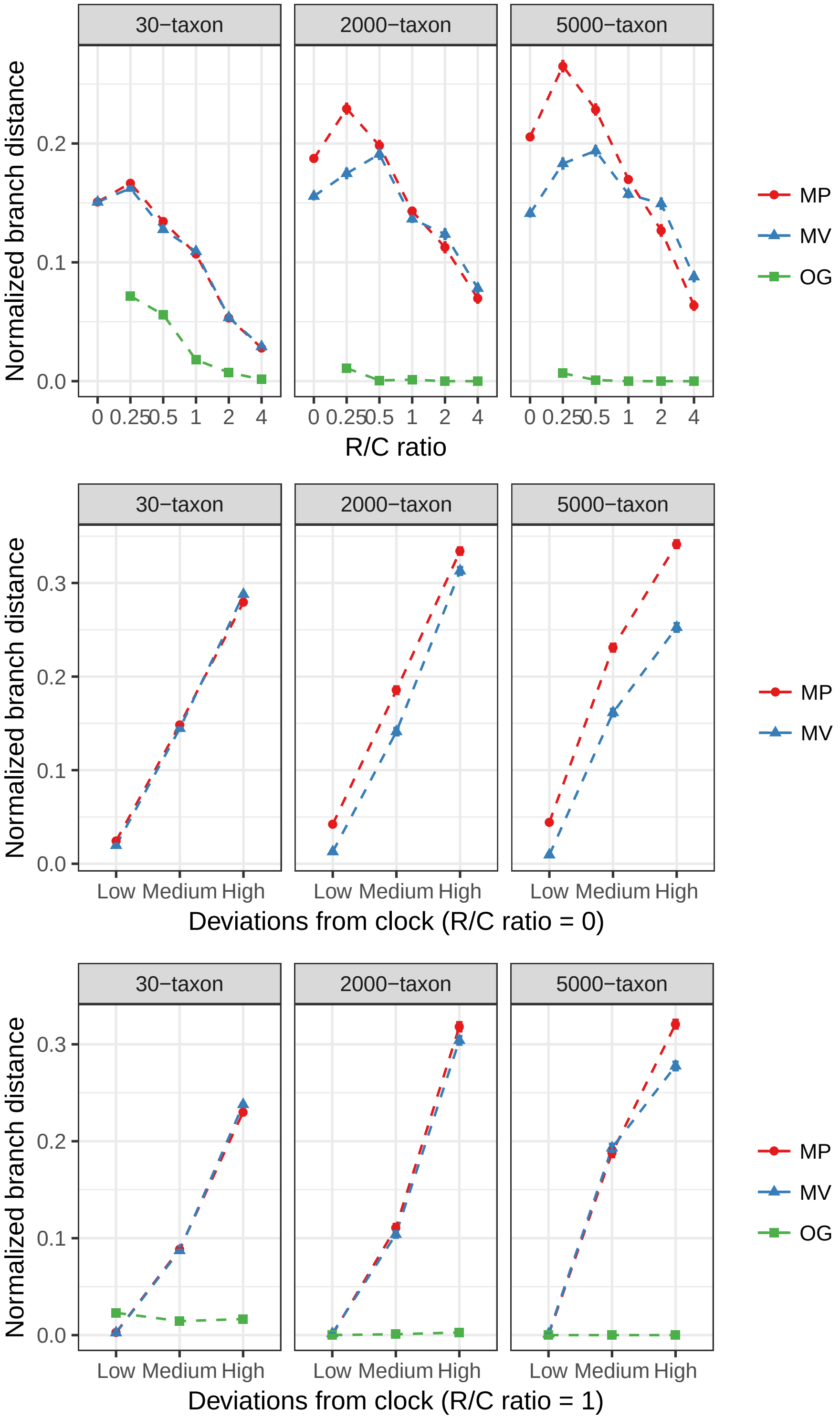

Fig D. Normalized branch distance in true rooted gene trees for datasets D1 and D2. The number of branches away from the true root is normalized by the tree depth and is shown for all three methods of rooting.

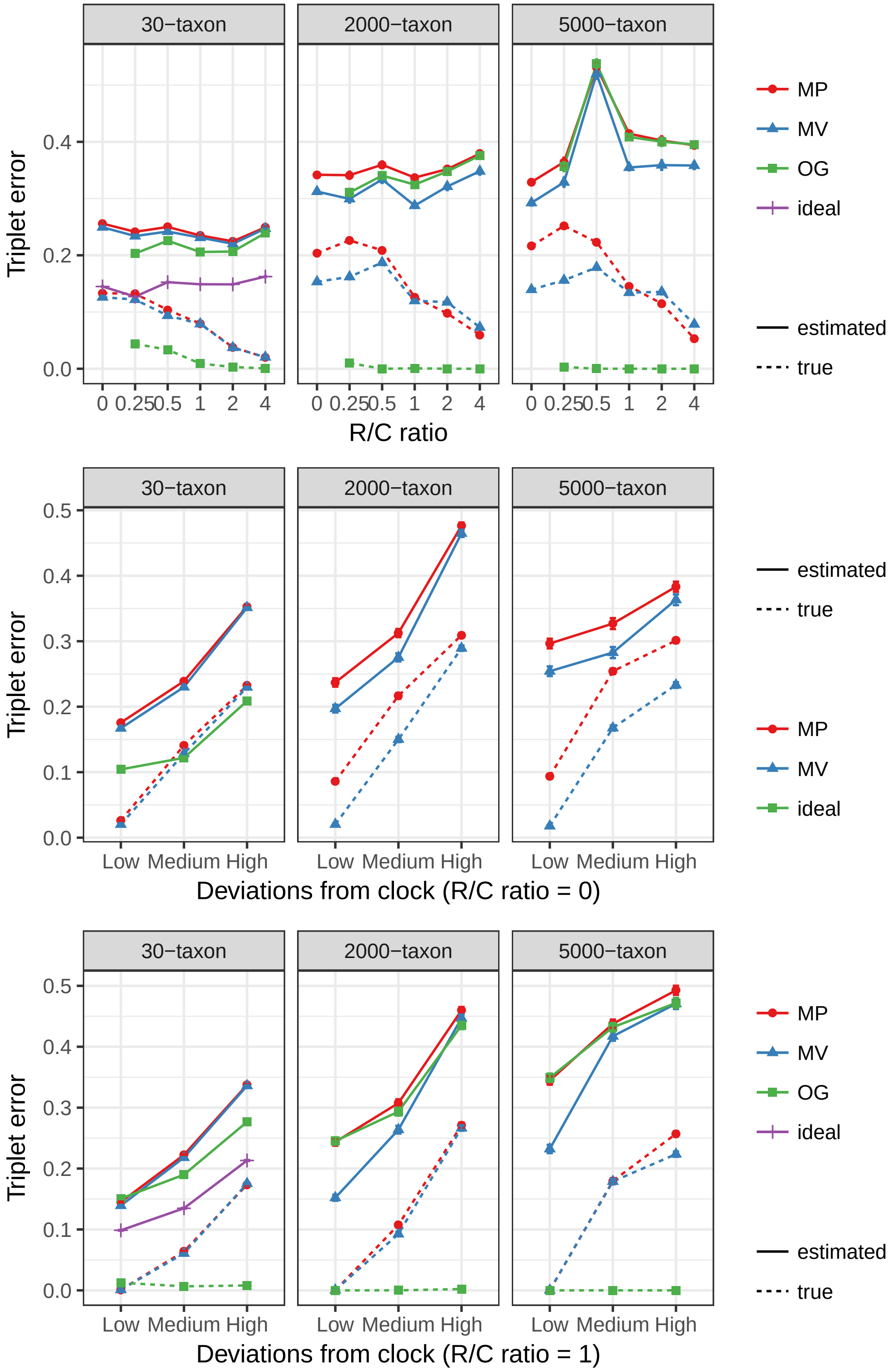

Fig E. Triplet error in true and estimated rooted gene trees for datasets D1 and D2. Absolute triplet distance is shown for all three methods of rooting plus the ideal rooting for D1 where a brute force calculation was feasible (the rooting that minimizes the triplet distance to the true tree).

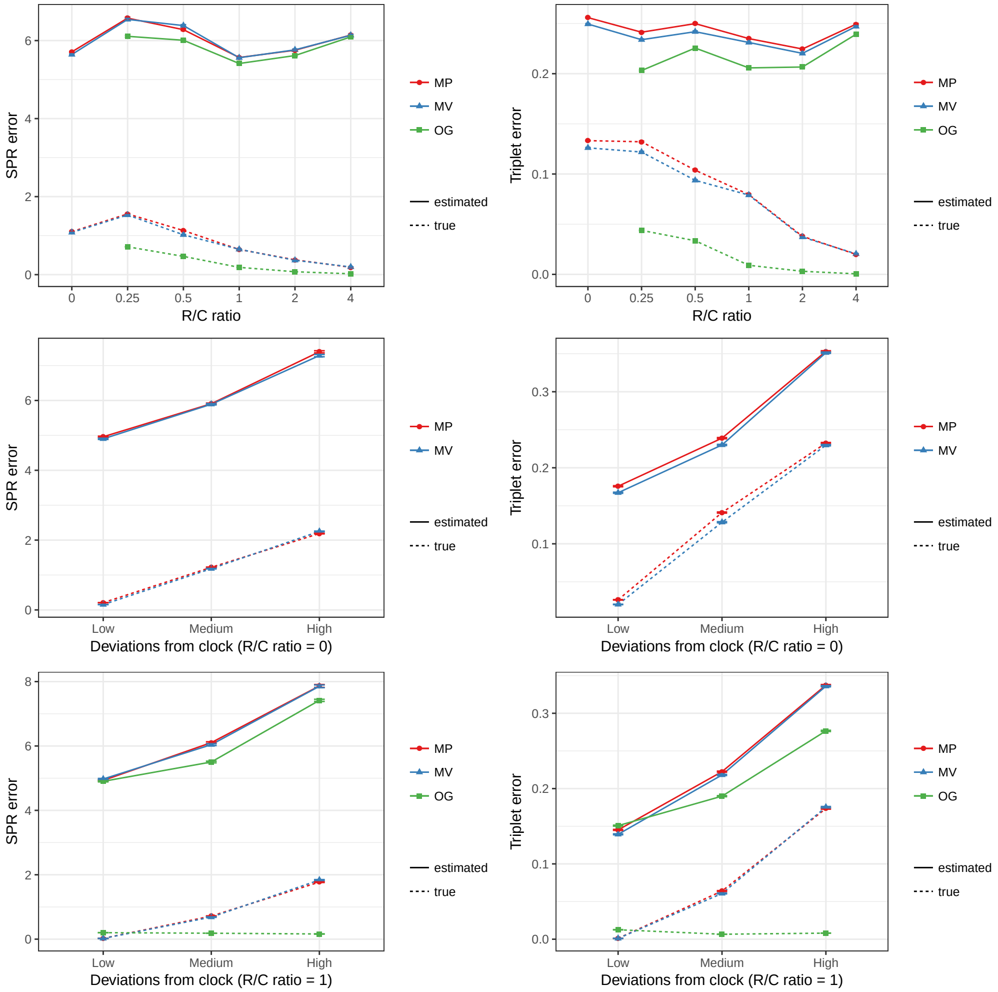

Fig F. SPR and Triplet error in true and estimated rooted gene trees for the 30-taxon dataset where SPR computation is feasible. Top: SPR and Triplet error with different R/C ratio. Middle and Bottom: SPR and Triplet error with different levels of deviations

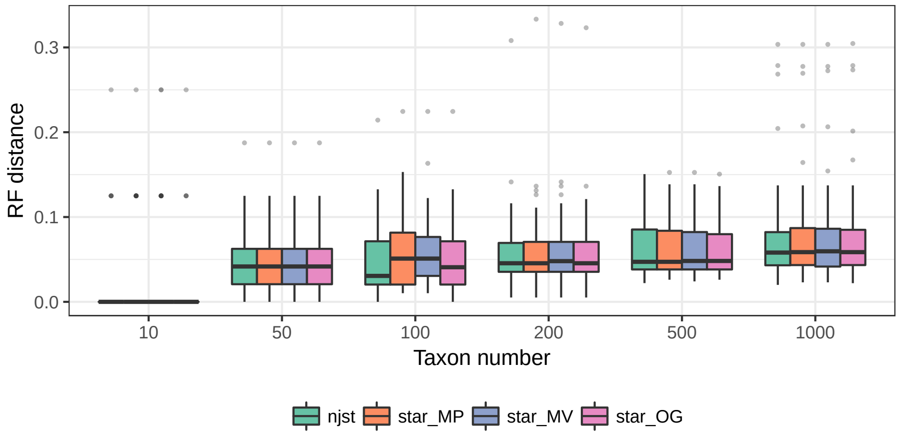

Fig G. STAR and NJst error on estimated gene trees for dataset D3. Species trees are estimated on estimated gene trees. RF distance is shown for NJst and STAR with all three methods of rooting.

Supplementary methods

Simulation setup

In order to simulate the gene sequences we used Indelible for datasets D1 and D2, with sequence lengths and mutation parameters drawn randomly from distributions described below. D1 has 30 taxa and D2 is a large dataset with 2000 or 5000 taxa. Note that in order to match the level of gene tree error observed in D1 in the D2 dataset, which included many more species, we set our sequence length hyperparameters such that we had longer sequence lengths in D2.

Gene Lengths: In D1, for each gene, we sample the sequence lengths from a log normal distribution. The parameters of the log normal (μ and σ) are drawn randomly from gamma and uniform distributions, respectively, for each individual replicate. We draw μ from a distribution because we want some replicates with high gene length (thus, low gene tree error) and some with low gene length. Similarly, we draw σ from a distribution to have replicates with high or low gene tree error variation.

Our goal was to have an average gene length of roughly 450 sites long across all datasets, which would lead to reasonable average levels of gene tree error. The σ parameter was drawn from a uniform random variable between (0.3,0.7) with the average of 0.5, and this range was empirically derived by trial and error. The mean of log-normal distribution is given by $e^{\mu+\sigma^2/2}$. For this number to be around 450, we need $\mu + \sigma^2/2 = \log(450)$. Replacing σ with its expected value, 0.5, we get that the expected value of μ should be $log(450) - 1/8$. The gamma distribution (which we use for μ) has an expected value of $shape \times scale$. We empirically observed that a scale of 0.033 results in sufficient variations. So in order to have the mean 450 for log-normal, we parameterize the gamma distribution with scale 0.033 and the shape $(log(450) - 1/8)/0.033$ and draw a value X from this distribution. This procedure gives us a left-skewed distribution with many numbers below 450. In order to make the distribution right-skewed (and avoid many genes with very few sites), we used a simple trick. We use $Y = 2\log(450) - 1/8 - X$ as our draw of μ. The expected value of Y remain $\log(450) - 1/8$, which in turn, leads to expected gene length of 450; however, the distribution becomes right-skewed. This gives us an empirical average sequence length of 495. The median sequence lengths is between 370 and 422 in 90% of replicates.

In D2, for each gene, we used the same strategy but with a target gene length of 700bp instead of 450bp (since larger trees need more sites to achieve similar accuracy). The rest of the procedure remains the same. The empirical average sequence length was 766, and the median sequence lengths was between 294 and 1236 in 90% of replicates.

Base frequencies: For both datasets D1 and D2 we used a Dirichlet(36 26 28 32) to draw base frequencies for A, C, G, and T. These values are ML estimates of the three previously published large biological datasets, and are obtained from a previous dataset [1].

Data availability

The code, datasets, and scripts used are all available at:
`https://uym2.github.io/MinVar-Rooting/`

References

1. Mirarab S, Warnow T. ASTRAL-II: coalescent-based species tree estimation with many hundreds of taxa and thousands of genes. Bioinformatics. 2015;31(12):i44–i52. doi:10.1093/bioinformatics/btv234.
